# The prognosis of non‐small cell lung cancer combined with chronic obstructive pulmonary disease: A systematic review and meta‐analysis

**DOI:** 10.1111/crj.13144

**Published:** 2020-02-03

**Authors:** Ke Wu, Jing Wang, Limin Zhao, Pei Wang, Qi Duan

**Affiliations:** ^1^ Department of Respiratory and Critical Care Medicine People's Hospital of Zhengzhou University Zhengzhou China; ^2^ Department of Respiratory Medicine People's Hospital of Henan University Zhengzhou China; ^3^ Department of Respiratory Medicine Henan Provincial People's Hospital, People's Hospital of Zhengzhou University Henan China; ^4^ Department of Respiratory Medicine The Fifth Affiliated Hospital Of Zhengzhou University Zhengzhou China

**Keywords:** chronic obstructive pulmonary disease, meta‐analysis, non‐small cell lung cancer, the prognosis

## Abstract

**Objective:**

To investigate the effect of non‐small cell lung cancer (NSCLC) combined with Chronic Obstructive Pulmonary Disease (COPD) on prognosis, so as to provide help to clinical diagnosis and treatment.

**Methods:**

PubMed, Web of Science and the Medline were used to retrieve studies reported from 1950 to 2019. If the study reported the prognosis of COPD with NSCLC, the study was included and the relevant information was extracted and the data analyzed. The standard mean deviation (SMD) of 95% confidence interval was used in this meta‐analysis.

**Results:**

A total of 851 data studies were reviewed for eligibility, eventually, 12 case‐control studies were included in this systematic review and meta‐analysis. The sample sizes for company's studies of NSCLC ranged from 85 to 10 378, with a total number of 14 164, including 2450 COPD and 9395 Non‐COPD. Meta‐analyses showed that concomitant COPD was associated with poorer OS compared with patients without COPD (Fixed: HR: 1.16; 95% CI: 1.08‐1.25. Random: HR: 1.07; CI: 0.92‐1.24), with a significant heterogeneity across studies (*I* ^2^ = 45%, *P* = 0.04).

**Conclusion:**

Our study showed that patients with combined COPD had an impact on the prognosis and survival rate of SCLC patients.

## 1 INTRODUCTION

Chronic obstructive pulmonary disease (COPD) and lung cancer (LC) are the two most common causes of death worldwide, killing 3 and 1.7 million people respectively each year. COPD is a common chronic airway disease in the respiratory system characterized by continuous airflow limitation. Its morbidity and mortality are increasing year by year, and it is expected to become the third leading cause of disease death in the world by 2020. LC is the most frequently diagnosed cancer worldwide and the leading cause of cancer‐related deaths, with a 5‐year survival rate of about 10% to 20%. It has become the fifth leading cause of death worldwide.

Chronic obstructive pulmonary disease and LC are related lung diseases. Studies have shown that they share the same risk factors, such as smoking and other inhaled substances, for occupational, household or outdoor air pollution. COPD is an important risk factor for the development of LC, and LC is also an important cause of death in COPD patients. In the diagnosis of LC, non‐small cell LC (NSCLC) patients account for 80%.^1^ COPD is considered to be one of the important comorbid diseases in NSCLC patients, with a prevalence of 50%‐70%.^2^, ^3^ In the past 30 years, although the association between COPD and LC risk has been well recognized, relatively little is known about the prognostic impact of COPD in patients with LC. Several studies have investigated the effect of COPD on survival of NSCLC, but the results are not clear.

This study aims to clarify the prognosis of patients with COPD combined with NSCLC, including mortality and survival rate, through a meta‐analysis.

## 2 Materials and methods

### 2.1 Search strategy

The primary sources for the literature search were the following electronic databases: PubMed, Web of Science and the Medline, Published between 1950 and 2019. And no language limitation was imposed during the retrieval. The search was limited by using the following search terms: (COPD or chronic obstructive pulmonary disease) AND (non‐small cell LC or NSCLC) AND (prognosis or prognostic or outcome or survival). The title and abstract of each identified study were scanned to exclude any irrelevant publications. The remaining articles were reviewed to determine whether they contained information on the topic of interest. We also supplemented this search by examining the reference lists of all of the retrieved publications and by identifying additional relevant articles.

### 2.2 Eligibility criteria

We formulated the following inclusion and exclusion criteria to determine the eligible studies included in our meta‐analysis.

Inclusion criteria:

1. studies compared patients with NSCLC with and without COPD;
2. LC was the study's primary disease;
3. the target outcomes were prognosis of COPD with NSCLC, including survival and mortality;
4. the hazard ratio (HR) with corresponding 95% confidence interval (CI) was validly reported in original literature or generated by sufficient data;

Exclusion criteria:

1. studies had no control patients;
2. studies cannot be found the full text;
3. poor literature quality;
4. letters, comments, review articles, conference proceedings, case‐reports and unpublished data.

We included more recent articles with the largest sample sizes to avoid overlapping patient data in duplicate publications, when there were duplicate publications.

### 2.3 Data extraction

Screening of potentially eligible studies was conducted independently by two authors, and disagreement being resolved by consensus. Baseline characteristics and outcomes were extracted: first author, publication year, study design, sample sizes included the number of total COPD with NSCLC and COPD, prognosis of patients in both groups.

### 2.4 Assessment of study quality

As for case‐control studies, we applied the Newcastle‐Ottawa Quality Assessment Scale, with a total score of 0‐3, 4‐6 and 7‐9 considered low, moderate and high quality, respectively. The quality of all studies was evaluated by a consensus meeting with all authors. Low‐quality studies were excluded from our meta‐analysis.

### 2.5 Statistical analysis

Review Manager 5.3 software was used to conduct statistical analysis on the strength of the association between NSCLC and COPD, and calculate the odd ratio (OR) of effect indicators and 95% confidence interval (95% CI) of each study. Heterogeneity test should be performed before merging effect size. When the heterogeneity test result is *P* > 0.10 and *I* ^2^ ≤ 50%, it can be considered that there is homogeneity among multiple similar studies. Fixed effect model is used to calculate the combined effect quantity, and the random effect model is used to calculate the combined effect size. The forest plot and funnel plot of meta‐analysis results were drawn.

## 3 Results

### 3.1 Literature search

**Figure 1:**
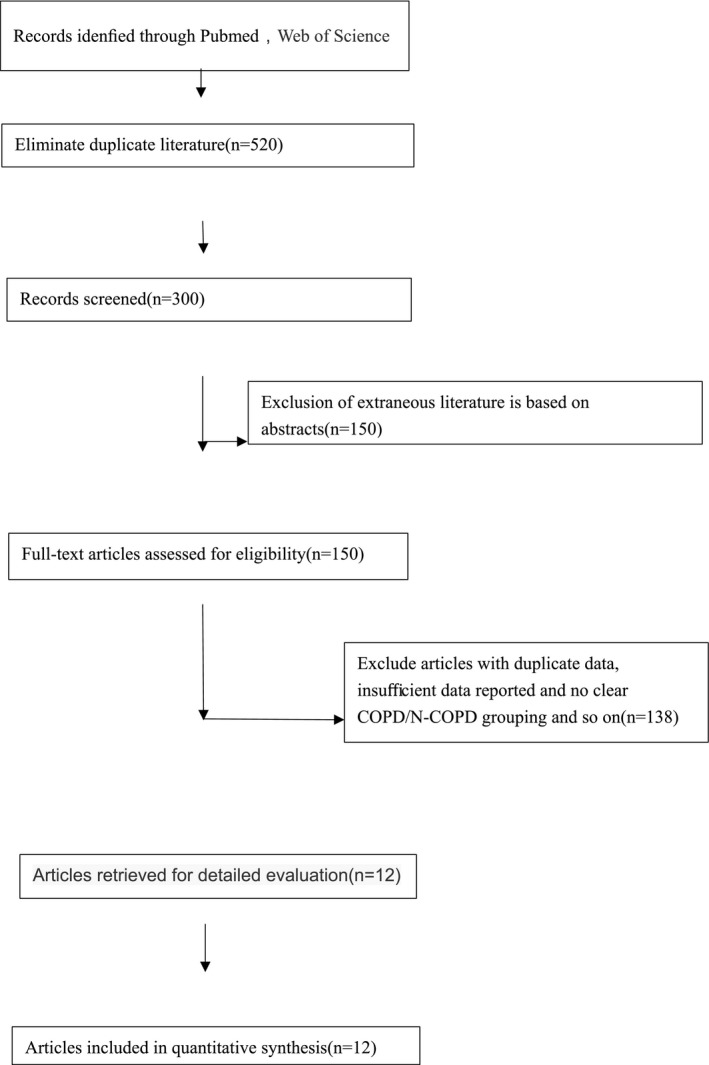
A flow chart showing the procedure for identifying the studies included in the meta‐analysis

Figure 1 presents the flowchart for identification of relevant studies. In total, we identified 851 records in the electronic databases. After initial screening based on titles and abstracts with predefined inclusion/exclusion criteria, 150 articles were identified for full‐text review. Eventually, 11 case‐control studies were included in this systematic review and meta‐analysis. Detailed characteristics of included studies are provided in Table 1.

**Table 1:** Characters of included studies

Study source	Study design	Study population			Gender (m/f)	Age, years, mean	Follow‐up, months	COPD ascertainment	Outcomes	HR estimation	Multivariate analysis
		NSCLC	COPD	Non COPD							
1 http://nc.yuntsg.com/pubmed/?term=Omote%2520N%255BAuthor%255D%26cauthor=true%26cauthor_uxml:id=29270008 4	CS	85	43	42	COPD: 37/6	COPD: 67 ± 8	NR	GOLD	OR	Reported in text	Yes
					Non‐COPD: 32/10	Non‐COPD: 63 ± 8					
2 Media Ara Shwan^5^	CS	534	329	141	COPD: 191/138	COPD: 69.4 ± 9.0	60	Medical record	5‐year survival	Reported in text	Yes
					Non‐COPD: 68/73	Non‐COPD: 65.9 ± 10.4		Spirometry			
3 Takaki Akamine^6^	CS	548	167	381	COPD: 129/38	Never smokers: 67.7 ± 9.9	38	Spirometry	RFS	Reported in text	Yes
					Non‐COPD: 185/196	Smokers: 67.9 ± 9.4			CSS		
									OS		
4 Nader Mina^7^	CS	107	88	19	NR	NR	12	Spirometry and/or CT	One/Two year survival rate	Reported in text	Yes
							24				
5 Seung Jun Lee^8^	CS	221	111	110	COPD: 97.3%/2.7%	COPD: 67.3 ± 8.0	24	GOLD	OS	Reported in text	Yes
					Non‐COPD: 96.4%/3.6%	Non‐COPD: 62.1 ± 10.3	60				
6 Ju S^9^	CS	110	57	53	COPD: 52/5	COPD: 67.5 ± 7.4	21	ATS	OS	Reported in text	Yes
					Non‐COPD: 44/9	Non‐COPD: 65.2 ± 7.8		ERS	PFS		
7 Maria Iachina^10^	CS	10 378	845	7278	COPD: 452/393	NR	NR	Medical record	survival	Reported in text	Yes
					Non‐COPD: 3568/3710						
8 Yi Young‐Soo^11^	CS	337	170	167	COPD: 154/16	COPD: 70.4 ± 8.9	25	GOLD	Quality of life survival	Reported in text	Yes
					Non‐COPD: 108/59	Non‐COPD: 63.2 ± 11.6					
9 Rihong Zhai^12^	CS	902	330	572	NR	COPD: 66.8 ± 9.7	60	Doctor‐diagnosis	OS	Reported in text	Yes
						Non‐COPD: 67.1 ± 10.1			PFS		
10 Lim Jeong Uk^13^	CS	345	68	277	COPD: 30/38	Median age: COPD75.2	NR	ATS	OS	Reported in text	Yes
					Non‐COPD: 54/223	Non‐COPD64.2		ERS			
11 J.A. Gullón^14^	CS	353	164	189	NR	NR	24	Spirometry	Survival	Reported in text	Yes
12 Yasuo Sekine^15^	CS	244	78	166	COPD: 58/20	COPD: 65.3 ± 7.9	24‐60	Spirometry	OS	Reported in text	No
					Non‐COPD: 95/71	Non‐COPD: 63.6 ± 9.7					

The sample sizes for different studies of NSCLC ranged from 85 to 10 378, with a total number of 14 164, including COPD 2450 and Non‐COPD 9395. 12 case‐control studies were conducted in Korea,^8^, ^9^, ^11^, ^13^ Denmark,^5^, ^9^ Japan,^4^, ^6^, ^15^ the United States^7^, ^12^ and Spain.^14^ In terms of diagnosis of COPD, 4 studies used Spirometry,^5^, ^6^, ^14^, ^15^ 3 studies used GOLD guideline,^4^, ^8^, ^11^ 2 studies used American Thoracic Society criteria or European Respiratory Society criteria,^9^, ^13^ 1 studies used Spirometry and/or Computed tomography (CT),^7^ 1 studies used Medical record^10^ and 1 studies used doctor‐diagnosis.^12^

As for quality assessment, the case‐control studies had a moderate to high quality (Newcastle‐Ottawa Quality Assessment Scale score 4‐9). Details of specific assessment are listed in Table 2.

**Table 2:** Assessment quality of included studies

Source	Choose				Comparability	Measurement of exposure factors			Score
	Adequate case definition	Represent‐ativeness of cases	Contrast to choose	Contrast to determine		Determination of exposure	The determination method of the exposed group was the same as that of the control group	No response rates	
1 http://nc.yuntsg.com/pubmed/?term=Omote%2520N%255BAuthor%255D%26cauthor=true%26cauthor_uxml:id=29270008 4	Independent verification	Yes	Hospital control	Without COPD	Control for age and other factors	Medical records	Yes	Not described	6
2 Media Ara Shwan^5^	Independent verification	Yes	Hospital control	Without COPD	Control for age and other factors	Medical records	Yes	Not described	6
3 Takaki Akamine^6^	Independent verification	Yes	Hospital control	Without COPD	Control for age and other factors	Medical records	Yes	Not described	6
4 Nader Mina^7^	Independent verification	Yes	Hospital control	Without COPD	Control for age and other factors	Medical records	Yes	Not described	6
5 Seung Jun Lee^8^	Independent verification	Yes	Community control	Without COPD	Did not control for age and other factors	Medical records	Yes	Not described	4
6 Ju S^9^	Independent verification	Yes	Hospital control	Without COPD	Control for age and other factors	Medical records	Yes	Not described	6
7 Maria Iachina^10^	Independent verification	Yes	Community control	Without COPD	Control for age and other factors	Medical records	Yes	Not the same	7
8 Yi Young‐Soo^11^	Independent verification	Yes	Hospital control	Without COPD	Control for age and other factors	Medical records	Yes	Not described	6
9 Rihong Zhai^12^	Independent verification	Yes	Hospital control	Without COPD	Control for age and other factors	Medical records	Yes	Not described	6
10 Lim Jeong Uk^13^	Independent verification	Yes	Hospital control	Without COPD	Control for age and other factors	Medical records	Yes	The same	7
11 J.A. Gullón^14^	Independent verification	Yes	Community control	Without COPD	Control for age and other factors	Medical records	Yes	Not described	7
12 Yasuo Sekine^15^	Independent verification	Yes	Hospital control	Without COPD	Control for age and other factors	Medical records	Yes	Not described	6

### 3.2 The prognosis of NSCLC with COPD

**Figure 2:**
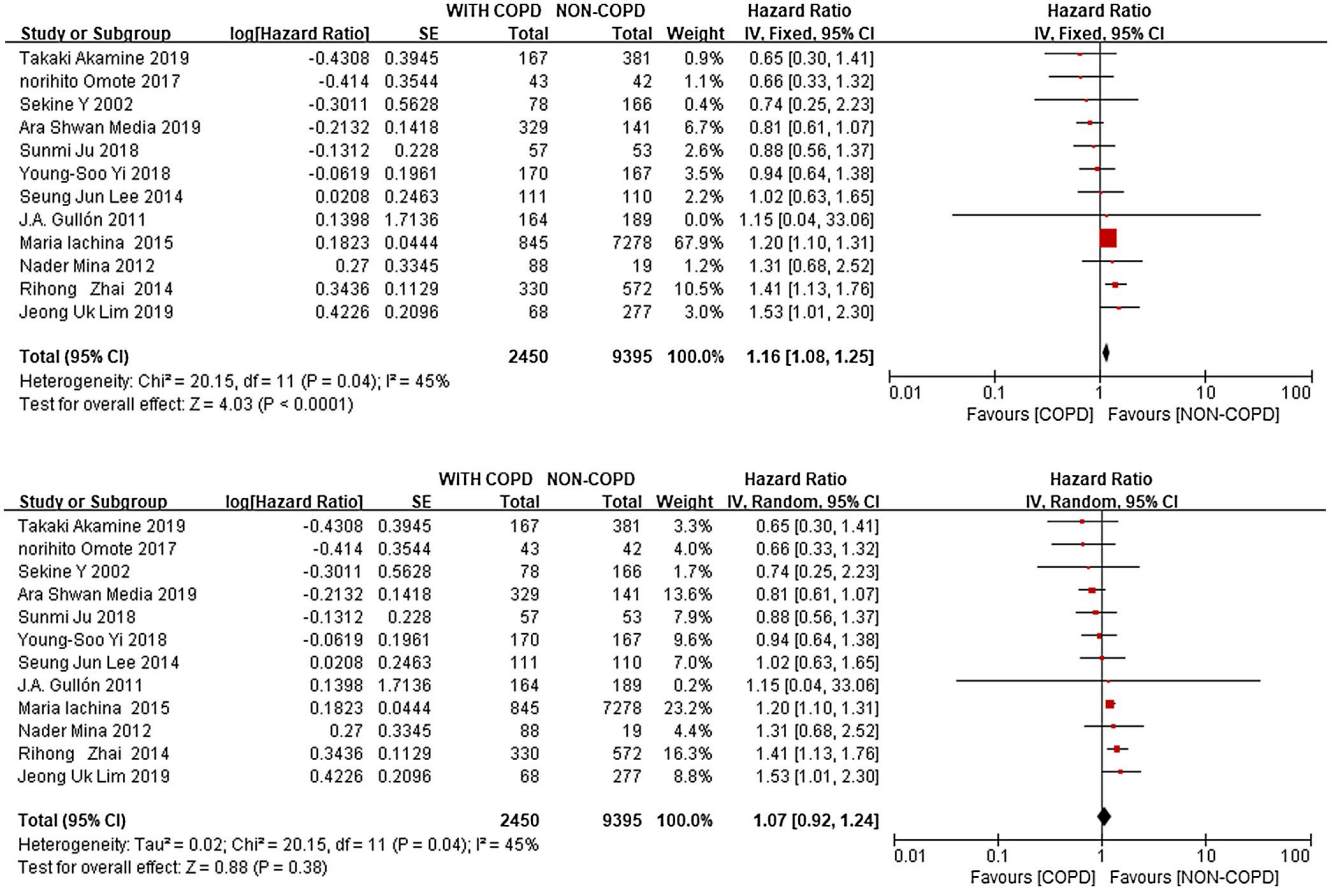
Forest plots of the associations between concomitant COPD and non‐small cell lung (the fixed effects model and random effects model)

A total of 12 studies evaluated the association between NSCLC and COPD. Meta‐analyses showed that concomitant COPD was associated with poorer OS compared with patients without COPD (Fixed: HR: 1.16; 95% CI: 1.08‐1.25. Random: HR: 1.07; CI: 0.92, 1.24) with a significant heterogeneity across studies (*I* ^2^ = 45%, *P* = 0.04) (Figure 2).

### 3.3 Publication bias

**Figure 3:**
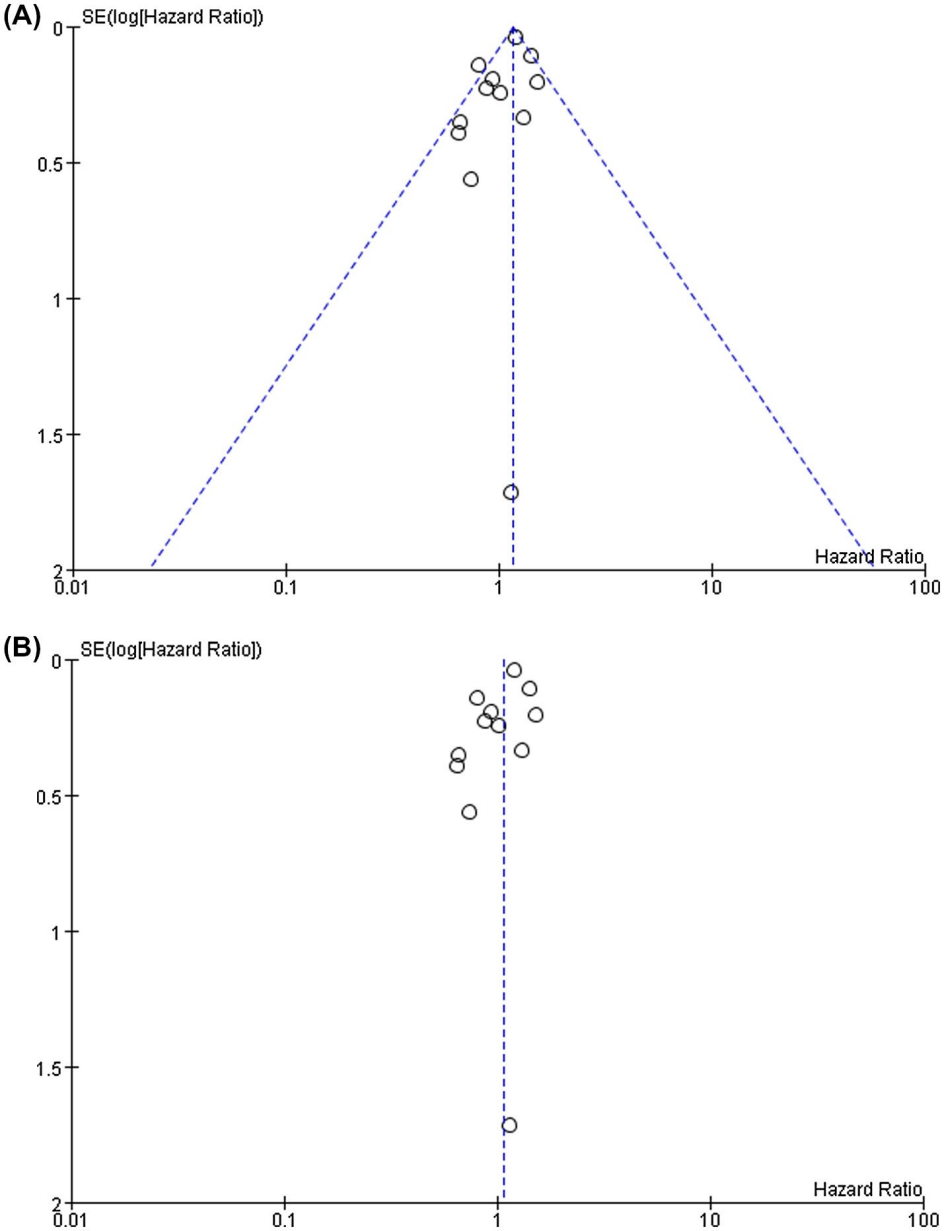
Funnel plots to assess publication bias regarding the association between concomitant chronic obstructive pulmonary disease and non‐small cell LC overall survival (A is the funnel plots corresponding to the fixed effect model; B is the funnel plots corresponding to the random effect model)

The left and right sides of the funnel plot are basically symmetric by visual inspection that indicated no significant publication bias was found in this meta‐analysis (Figure 3).

## 4 DISCUSSION

Our meta‐analysis based on the prognosis of NSCLC with COPD showed that NSCLC patients with COPD were associated with poor prognosis (Fixed: HR: 1.16; 95%CI: 1.08‐1.25. Random: HR: 1.07; CI: 0.92‐1.24), with a significant heterogeneity between studies (*I* ^2^ = 45%, *P* = 0.04). It suggests that the prognosis of patients with NSCLC complicated with COPD is poor, and the survival rate is reduced and the mortality rate is increased. Previous studies have found that COPD is an important factor in the occurrence and development of LC. Therefore, in the diagnosis and treatment of NSCLC, attention should be paid to the screening of coexisting COPD, so as to early detection and treatment and improve the prognosis of patients with NSCLC complicated with COPD.

The prognosis of LC is poor, and the 5‐year survival rate is about 10%‐15%. In the diagnosis of LC, NSCLC is the main one. The number of new cases is expected to increase as the population ages, and LC patients will increasingly coexist with the disease. As another common respiratory disease, COPD, the incidence and mortality are also increasing year by year, and it is also one of the important comorbid diseases of LC patients, which has been widely reported as an important factor in the occurrence and development of LC.^16^, ^17^, ^18^ At present, there are many reports on the impact of COPD on the prognosis of NSCLC, but opinions are not uniform. Maria Iachina et al. showed that the survival rate of NSCLC patients with COPD significantly decreased,^6^, ^10^, ^11^, ^12^, ^13^, ^14^, ^15^ and Norihito Omote et al showed that mild to moderate COPD had no significant adverse effect on the prognosis of NSCLC patients.^4^, ^5^, ^7^, ^8^, ^9^ Our meta‐analysis showed that NSCLC with COPD had a poor prognosis, similar to the results of Maria Iachina et al. Besides, Maria Iachina contributed significantly to the study, accounting for 67.9% (Fixed effect model), which seemed to be related to the large proportion of the total sample size included and the large proportion of the sample size of NSCLC without COPD.

The fixed effects model of meta‐analysis is based on the assumption that a number of studies have a common effect scale, and the difference in the effect scale of each study is mainly caused by random error. However, the random effects model assumes that each study does not have a common effect scale, but each study has its own effect scale, and a balance is obtained by using the inter‐group and intra‐group differences. In our study, we included studies from different parts of the world with very different survival environments and demographic characteristics, as well as different rates of COPD and NSCLC. In this study, the fixed effects model and the random effects model have the same *P* value and *I* ^2^ value. However, it is not appropriate to use the same effect scale in the fixed effects model to measure these studies, for example, the contribution of J.A. Gullon’s research is ignored in the solid effects model that we adopted. Therefore, the random effects model seems to be more appropriate, which allows the heterogeneity of each included sample, and the weight of each sample is more suitable for this study than the fixed effect model. However, several limitations should be taken into account when interpreting our study. First, there are relatively few participants. The total is still not large enough to reduce statistical significance; Second, we failed to include high‐quality randomized controlled trials; Third, no further analysis was conducted on the effects of different grades of COPD on the prognosis of NSCLC and on the prognosis of different stages of NSCLC. Fourth, this study did not search the Chinese literature, which may exclude several studies aimed at exploring the relationship between COPD and NSCLC in Chinese.

## CONFLICT OF INTEREST

The authors have declared that there is no conflict of interest.

## AUTHOR CONTRIBUTIONS

All the authors revised and accepted the final version of the manuscript.

*Study design:* Wu, Zhao

*Manuscript writing:* Wu

*Data analysis:* Wang, Duan

## ETHICS

All analyses were based on previous published studies, thus no ethical approval and patient consent are required.
